# Conditional t-SNE: more informative t-SNE embeddings

**DOI:** 10.1007/s10994-020-05917-0

**Published:** 2020-12-06

**Authors:** Bo Kang, Darío García García, Jefrey Lijffijt, Raúl Santos-Rodríguez, Tijl De Bie

**Affiliations:** 1 Department of Electronics and Information Systems, IDLab, Ghent University, Ghent, Belgium; 2 Facebook AI, New York, USA; 3 Department of Engineering Mathematics, University of Bristol, Bristol, UK

**Keywords:** Dimensionality reduction, Information theory, Data visualization

## Abstract

Dimensionality reduction and manifold learning methods such as t-distributed stochastic neighbor embedding (t-SNE) are frequently used to map high-dimensional data into a two-dimensional space to visualize and explore that data. Going beyond the specifics of t-SNE, there are two substantial limitations of any such approach: (1) not all information can be captured in a single two-dimensional embedding, and (2) to well-informed users, the salient structure of such an embedding is often already known, preventing that any real new insights can be obtained. Currently, it is not known how to extract the remaining information in a similarly effective manner. We introduce *conditional t-SNE* (ct-SNE), a generalization of t-SNE that discounts prior information in the form of labels. This enables obtaining more informative and more relevant embeddings. To achieve this, we propose a conditioned version of the t-SNE objective, obtaining an elegant method with a single integrated objective. We show how to efficiently optimize the objective and study the effects of the extra parameter that ct-SNE has over t-SNE. Qualitative and quantitative empirical results on synthetic and real data show ct-SNE is scalable, effective, and achieves its goal: it allows complementary structure to be captured in the embedding and provided new insights into real data.

## Introduction

Dimensionality reduction (DR) methods can be used to create low-dimensional embeddings, e.g., two-dimensional (2D) embeddings that allow visualization of high-dimensional data and subsequently can be used to explore the high-level structure of such data. Non-linear DR methods are particularly powerful because they can capture complex structure even when it is spread over many dimensions. This explains the huge popularity of methods such as t-SNE (van der Maaten and Hinton 2008), LargeVis (Tang et al. 2016), and UMAP (McInnes and Healy 2018).

Yet, there are clear limitations to this approach using any existing DR method. Current methods yield a single static embedding, which is insufficient because (a) the most prominent structure present in the data may already be known to the analyst and (b) because a single 2D embedding typically cannot capture all structure present in the data. One may indeed construct higher-dimensional embeddings, hoping to uncover more structure. However, it is not obvious how to explore high-dimensional embeddings and there is no guarantee any 2D view of such an embedding would be unaffected by the previously known information. For the latter problem, one could consider removing all related attributes, but the known salient structure may indeed be spread across all attributes. Therefore, the question arises: *can we actively filter or discount prior knowledge from the embedding?*

To this end, we introduce *conditional t-SNE* (ct-SNE), a generalization of t-SNE that discounts prior information. By discounting prior information, the embedding may focus on capturing *complementary* information. Discounting here means that we value information that aligns with our expectations—that same-labelled points have high similarity—less than information contradicting our expectations. Concretely, ct-SNE does not aim to construct an embedding that reflects all pairwise proximities in the original data (which is the objective of t-SNE), but it should reflect each pairwise proximity *conditioned on whether we expect that pair to be close or not*, given the prior information.

ct-SNE enables at least three new ways to obtain insight into data:

- When prior knowledge is available beforehand, we can straight away focus the analysis on an embedding that is more informative.
- Such prior knowledge may be gained during analysis, leading to an iterative data analysis process and enabling deeper exploration of data.
- We can encode some information Y as prior information to test whether an observed effect X is complementary to Y. If X is factored out when Y is considered as prior, there is a dependancy, if X remains present, the effects are complementary.

Note we use the term *prior knowledge*, even when this knowledge is not available a priori, but gained during the analysis. This reflects the knowledge that is available just prior to the embedding step.

### Example

**Fig. 1:**
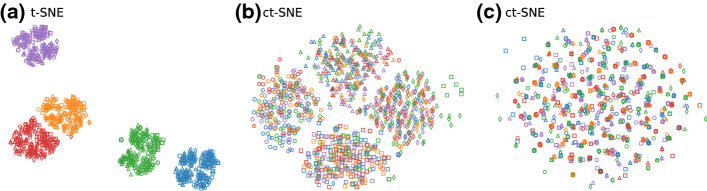
Visualization of 2-d embeddings of synthetic data. Colors and markers explained in ‘Example’ below (Color figure online)

To demonstrate the idea behind ct-SNE more concretely, consider a ten-dimensional dataset with 1000 data points. In dimension 1–4 the data points fall into five clusters (following a multi-variate Gaussian with small variance; the data generating process is explained in more detail in Sect. 3), similarly for dimensions 5–6 the points fall randomly into four clusters. Dimensions 7–10 contain Gaussian noise with larger variance. Figure 1a gives the t-SNE embedding. It shows five large clusters (colored), where some can be visibly somewhat clearly split further into smaller clusters. The large clusters correspond to those defined in dimension 1–4. Figure 1b is the ct-SNE embedding where we have input the five colored clusters as prior knowledge. This figure shows four clusters that are complementary to the five clusters observed in Fig. 1a. We see they are complementary because there is no correlation between the colors and the clusters in Fig. 1b. These four clusters are indeed those defined in dimensions 5–6. Notice that it is not possible to observe these four clusters (the ground truth clustering in dim. 5–6 is coded with marker shapes) as being coherent clusters in Fig. 1a. The four clusters of Fig. 1b are spread over the five colored clusters of Fig. 1a. Finally, Fig. 1c shows that after combining the labels as prior knowledge, ct-SNE yields an embedding capturing only on the remaining noise. The lack of visible structure aligns with the data not having any structure beyond the now encoded prior knowledge.

### Contributions

This paper contributes the following:

- The introduction of ct-SNE, a new DR method that searches for an embedding such that a distribution defined in terms of distances in the input space (as done in t-SNE) is well-approximated by a distribution defined in terms of distances in the embedding space *after conditioning on the prior knowledge*;
- A Barnes-Hut-Tree based optimization method to efficiently find a good embedding;
- An illustration of how this concept of conditioning embeddings can be used with other DR methods;
- Qualitative and quantitative experiments on synthetic and real-world datasets, which show that ct-SNE (1) effectively removes given prior information, (2) enables more in-depth visual analysis of high-dimensional data, and (3) scales sufficiently to handle hundreds of thousands of points.

The implementation of ct-SNE and the code for experiments on public data are available at https://bitbucket.org/ghentdatascience/ct-sne.

### Structure

Section 2 introduces the method; experiments are presented in Sect. 3, and related work is discussed in Sect. 4. Section 5 concludes the paper.

## Method

In this section, we derive ct-SNE and describe a Barnes-Hut based strategy to optimize the ct-SNE objective. We begin with a recap of t-SNE.

### Background: t-SNE

In t-SNE, two conditional distributions for the pairwise similarity in the original high-dimensional space, $p_{ij}$, and the low-dimensional approximation, $q_{ij}$, are posited. The goal is to optimize the embedding—which affects the $q_{ij}$ probabilities—such that $p_{ij}$ and $q_{ij}$ are as similar as possible, the difference being quantified by KL divergence.

One way to interpret these conditional probabilities is as follows: The input data ${\varvec{X}}\in {\mathbb {R}}^{n\times d}$ defines a probability distribution for a categorical random variable *e*, of which the value domain is indexed by all pairs (*i*, *j*) with $i,j\in [1..n]$ and $i\ne j$. This distribution is determined by probabilities $0\le p_{ij}\le 1$ s.t. $\sum _{i, j} p_{ij}=1$. Each $p_{ij}$ equals the probability that $e=(i,j)$. For brevity, we speak of *the distribution* ${\varvec{p}}$ when we mean the categorical distribution with parameters $p_{ij}$.

In t-SNE, the distribution ${\varvec{p}}$ is defined as follows:

1 $$\begin{aligned} p_{ij}&\triangleq P_{\varvec{p}}(e=(i,j))\nonumber \\&= \frac{1}{2n}\left( \frac{\exp \left( -\Vert {\varvec{x}}_i-{\varvec{x}}_j\Vert ^2/2\sigma _i^2\right) }{\sum _{k\ne i}\exp \left( -\Vert {\varvec{x}}_i-{\varvec{x}}_k\Vert ^2/2\sigma _i^2\right) }+ \frac{\exp \left( -\Vert {\varvec{x}}_j-{\varvec{x}}_i\Vert ^2/2\sigma _j^2\right) }{\sum _{l\ne j}\exp \left( -\Vert {\varvec{x}}_j-{\varvec{x}}_l\Vert ^2/2\sigma _j^2\right) }\right) \end{aligned}$$

where $\sigma _i$ is obtained by performing a binary search for the value of $\sigma$s that produces a distribution $\frac{\exp \left( -\Vert {\varvec{x}}_i-{\varvec{x}}_j\Vert ^2/2\sigma _i^2\right) }{\sum _{k\ne i}\exp \left( -\Vert {\varvec{x}}_i-{\varvec{x}}_k\Vert ^2/2\sigma _i^2\right) }$ with a fixed perplexity^1^ that is specified by the user. The first term of Eq. (1) transforms the distance from data point ${\varvec{x}}_i$ to data point ${\varvec{x}}_j$ in the input representation using a Gaussian density function centered at ${\varvec{x}}_i$. For nearby data points, the density value is relatively high, whereas for data points that are far apart, the density value is very small. However, because of the quick vanishing of density values, the outlier points will be randomly placed in the lower dimensional embedding. Adding the second term of Eq. (1) as the Gaussian density function centered at ${\varvec{x}}_j$ symmetrizes the similarity measure between the input data points, which allows each data point (including outliers) to equally contribute to the cost function. This gives a better lower dimensional representation when outliers are present.

The goal of t-SNE is to find another embedding ${\varvec{Y}}\in {\mathbb {R}}^{n\times d^{\prime}}$, from which another categorical probability distribution is derived, specified by the values $q_{ij}$:

2 $$\begin{aligned} q_{ij}\triangleq P_{\varvec{q}}e=(i,j))=\frac{(1+\Vert {\varvec{y}}_i-{\varvec{y}}_j\Vert ^2)^{-1}}{\sum _{k\ne l}(1+\Vert {\varvec{y}}_k-{\varvec{y}}_l\Vert ^2)^{-1}}. \end{aligned}$$

An embedding ${\varvec{Y}}$ is deemed better if the distance between these two categorical distributions is smaller, as quantified by the KL-divergence: $KL({\varvec{p}}\Vert {\varvec{q}})=\sum _{i\ne j} p_{ij}\log \left( \frac{p_{ij}}{q_{ij}}\right) .$ By minimizing the KL-divergence, similar data points attract each other and dissimilar data points repel each other. This forms a low-dimensional representation that reflects the pairwise similarity in the input representation.

### Conditional t-SNE

Due to stochasticity in the optimization, each rerun of t-SNE produces a different embedding ${\varvec{Y}}$. However, the global structure of the embeddings is very similar, aiming to convey the original distances $\Vert {\varvec{x}}_i-{\varvec{x}}_j\Vert ^2$ as well as possible. However, as also highlighted in the example in Sect. 1, a 2D embedding can typically only capture part of the structure present in the data. For expert users, the captured dominant structure is also often already known. Hence, it is useful to factor out ‘prior knowledge’ from the embedding such that it can reveal other more fine-grained structure.

We achieve this as follows. For simplicity of presentation, assume that each data point ${\varvec{x}}_i$ has a label $l_i$, with $l_i\in [0..L]$ for all $i\in [1..n]$. Moreover, let us assume that we expect same-labeled data points more likely to be nearby each other in ${\varvec{X}}$. Our goal is to allow the embedding ${\varvec{Y}}$ not to reflect that information again. This can be achieved by minimizing the KL-divergence between the distributions ${\varvec{p}}$ and ${\varvec{r}}$ (rather than ${\varvec{q}}$), where ${\varvec{r}}$ is the distribution derived from the embedding ${\varvec{Y}}$ but *conditioned on the prior knowledge*.

We formalize this using the following notation. The indicator variable $\delta _{ij}=1$ if $l_i=l_j$ and $\delta _{ij}=0$ if $l_i\ne l_j$, and the label matrix ${\varvec {\Delta }}$ is defined by $[{\varvec {\Delta }}]_{ij}=\delta _{ij}$. Actually ${\varvec {\Delta }}$ can be any binary matrix, but for simplicity here it has block structure, being induced by a single categorical label for all data points. The probability that the random variable *e* is equal to (*i*, *j*), *conditioned on* the label matrix ${\varvec {\Delta }}$ (i.e. the prior information) is denoted as:

$$\begin{aligned} r_{ij}&\triangleq P_{\varvec{q}}(e=(i,j)|{\varvec {\Delta }})= \frac{P({\varvec {\Delta }}|e=(i,j))P_{\varvec{q}}(e=(i,j))}{P_{\varvec{q}}({\varvec {\Delta }})}. \end{aligned}$$

In ct-SNE, the embedding should be such that ${\varvec{r}}$ is similar to ${\varvec{p}}$. Note that if we ensure that $P({\varvec {\Delta }}|e=(i,j))$ is larger when $\delta _{ij}=1$ than when $\delta _{ij}=0$, it will be less important for the embedding to ensure that $q_{ij}=P_{\varvec{q}}(e=(i,j))$ is large for same-labeled data points, even if $p_{ij}$ is large. I.e., *for same-labeled data points*, it is less important to be embedded nearby even if they are nearby in the input representation.

To compute $P_{\varvec{q}}(e=(i,j)|{\varvec {\Delta} })$, we now investigate its different factors. First, $P_{\varvec{q}}(e=(i,j))=q_{ij}$ is simply computed as in Eq. (2). Second, we need to determine a suitable form for $P({\varvec {\Delta }}|e=(i,j))$. As motivated previously, $P({\varvec {\Delta }}|e=(i,j))$ should depend on the variable $\delta _{ij}$, indicating whether data points *i* and *j* share the same label. No additional functional dependency of $P({\varvec {\Delta }}|e=(i,j))$ on ${\varvec {\Delta }}$ is required for our purposes, nor would one be naturally justifiable. Thus, $\delta _{ij}$ is taken to be the sufficient statistic for $P({\varvec {\Delta }}|e=(i,j)),$ such that we can write $P({\varvec {\Delta }}|e=(i,j))=\alpha ^{\delta _{ij}}\beta ^{1-\delta _{ij}}$, where $\alpha$ and $\beta$ determine the probability of points ${\varvec{x}}_i$ and ${\varvec{x}}_j$ being randomly picked to have the same or different labels. Let us further denote the class size of the *l*’th class as $n_l$. Then, for this distribution to be normalized, it must hold that:

$$\begin{aligned} 1&= \sum _{{\varvec {\Delta }}} P({\varvec {\Delta }}|e=(i,j)) = \alpha \left( \sum _l\frac{(n-2)!}{(n_l-2)!\prod _{f\ne l}n_f!}\right) \\&\quad+ \beta \left( \frac{n!}{\prod _l n_l!}-\sum _l\frac{(n-2)!}{(n_l-2)!\prod _{f\ne l}n_f!}\right) \\&= \frac{n!}{\prod _l n_l!}\left( \alpha \frac{\sum _l n_l(n_l-1)}{n(n-1)}+\beta \left( 1-\frac{\sum _l n_l(n_l-1)}{n(n-1)}\right) \right) . \end{aligned}$$

This yields a relation between $\alpha$ and $\beta$. It also suggests a ballpark figure for $\alpha$. Indeed, one would typically set $\alpha >\beta$.^2^ For $\alpha =\beta$ (i.e. the lower bound for $\alpha$), they would both be equal to $\alpha =\beta =\frac{\prod _l n_l!}{n!}$, i.e. one divided by the number of possible distinct label assignments (which is logical). Thus, in tuning $\alpha$, one could take multiples of this minimal value.

We can now also compute the marginal probability $P_{\varvec{q}}({\varvec {\Delta }})$ as follows:

$$\begin{aligned} P_{\varvec{q}}({\varvec {\Delta }})&= \sum _{i\ne j} P({\varvec {\Delta }}|e=(i,j))P_{\varvec{q}}(e=(i,j))\\&= \sum _{i\ne j} q_{ij}\alpha ^{\delta _{ij}}\beta ^{1-\delta _{ij}}\\&= \alpha \sum _{i\ne j:\delta _{ij}=1}q_{ij} + \beta \sum _{i\ne j:\delta _{ij}=0}q_{ij}. \end{aligned}$$

Given all this, one can then compute the required conditional distribution as follows:

3 $$\begin{aligned} r_{ij}&\triangleq P_{\varvec{q}}(e=(i,j)|{\varvec {\Delta }}) = \frac{P({\varvec {\Delta }}|e=(i,j))P_{\varvec{q}}(e=(i,j))}{P_{\varvec{q}}({\varvec {\Delta} })}\nonumber \\&= \left\{ \begin{array}{ll} \frac{\alpha q_{ij}}{\alpha \sum _{i\ne j:\delta _{ij}=1}q_{ij} + \beta \sum _{i\ne j:\delta _{ij}=0}q_{ij}}&\quad {\text{ if }}\,\,\delta _{ij}=1,\\ \frac{\beta q_{ij}}{\alpha \sum _{i\ne j:\delta _{ij}=1}q_{ij} + \beta \sum _{i\ne j:\delta _{ij}=0}q_{ij}}&\quad {\text{ if }}\,\,\delta _{ij}=0. \end{array} \right. \end{aligned}$$

It is numerically better to express this in terms of new variables $\alpha ^{\prime}\triangleq \alpha \frac{n!}{\prod _l n_l!}$ and $\beta ^{\prime}\triangleq \beta \frac{n!}{\prod _l n_l!}$:

$$\begin{aligned} r_{ij}&= \left\{ \begin{array}{ll} \frac{\alpha ^{\prime} q_{ij}}{\alpha ^{\prime}\sum _{i\ne j:\delta _{ij}=1}q_{ij} + \beta ^{\prime}\sum _{i\ne j:\delta _{ij}=0}q_{ij}}&\quad {\text{ if }}\,\,\delta _{ij}=1,\\ \frac{\beta ^{\prime} q_{ij}}{\alpha ^{\prime}\sum _{i\ne j:\delta _{ij}=1}q_{ij} + \beta ^{\prime}\sum _{i\ne j:\delta _{ij}=0}q_{ij}}&\quad {\text{ if }}\,\,\delta _{ij}=0, \end{array} \right. \end{aligned}$$

where the relation between $\alpha ^{\prime}$ and $\beta ^{\prime}$ is:

4 $$\begin{aligned} 1 = \alpha ^{\prime}\frac{\sum _l n_l(n_l-1)}{n(n-1)}+\beta ^{\prime}\left( 1-\frac{\sum _l n_l(n_l-1)}{n(n-1)}\right) . \end{aligned}$$

This has the advantage of avoiding large factorials and resulting numerical problems. The lower bound for $\alpha ^{\prime}$ that can be considered is now 1 (then also $\beta ^{\prime}=1$).

Finally, computing the KL-divergence with ${\varvec{p}}$, yields the ct-SNE objective function to be minimized:

5 $$\begin{aligned} KL({\varvec{p}}\Vert {\varvec{r}})&= \sum _{i\ne j} p_{ij}\log \left( \frac{p_{ij}}{r_{ij}}\right) \end{aligned}$$

6 $$\begin{aligned}&= KL({\varvec{p}}\Vert {\varvec{q}}) + \sum _{i\ne j}p_{ij}\log \left( \frac{\alpha ^{\prime}\sum _{i\ne j:\delta _{ij}=1}q_{ij} + \beta ^{\prime}\sum _{i\ne j:\delta _{ij}=0}q_{ij}}{{\alpha ^{\prime}}^{\delta _{ij}}{\beta ^{\prime}}^{1-\delta _{ij}}}\right) \end{aligned}$$

7 $$\begin{aligned}&= KL({\varvec{p}}\Vert {\varvec{q}}) \end{aligned}$$

8 $$\begin{aligned}&+ \log \left( \alpha ^{\prime}\sum _{i\ne j:\delta _{ij}=1}q_{ij} + \beta ^{\prime}\sum _{i\ne j:\delta _{ij}=0}q_{ij}\right) \nonumber \\&\quad - \sum _{i\ne j:\delta _{ij}=1}p_{ij}\log (\alpha ^{\prime}) - \sum _{i\ne j:\delta _{ij}=0}p_{ij}\log (\beta ^{\prime}). \end{aligned}$$

Note that the last two terms are constant w.r.t. $q_{ij}$. Moreover, for $\alpha ^{\prime}=\beta ^{\prime}=1$, this equals standard t-SNE. For $\alpha ^{\prime}>1>\beta ^{\prime}$ (and subject to Eq. 4), the minimization of this KL-divergence will try to minimize $q_{ij}$ more strongly for *i*, *j* where $\delta _{ij}=1$ than when $\delta _{ij}=0$.

### Optimization

The objective function (Eq. 5) is not convex with respect to the embedding ${\varvec{Y}}$. Even so, we found that optimizing the objective function using gradient descent with random restarts works well in practice. The gradient of the objective function with respect to the embedding of a point ${\varvec{y}}_i$ reads:

$$\begin{aligned} \nabla _{{\varvec{y}}_i}KL({\varvec{p}}\Vert {\varvec{r}})&= 4\left( F_{\text {attr}} + F_{\text {rep}}\right) \\&= 4\sum _j\Big ( p_{ij}q_{ij}Z({\varvec{y}}_i - {\varvec{y}}_j) - \frac{\delta _{ij}\alpha ^{\prime} + (1-\delta _{ij})\beta ^{\prime}}{O}\cdot q_{ij}^2Z({\varvec{y}}_i - {\varvec{y}}_j)\Big ) . \end{aligned}$$

where $O = \alpha ^{\prime} \sum _{i\ne j: \delta _{kl} = 1}q_{kl} + \beta ^{\prime}\sum _{i\ne j: \delta _{kl} = 0}q_{kl}$ and $Z = \sum _{k\ne l} (1+\Vert {\varvec{y}}_k-{\varvec{y}}_l\Vert ^2)^{-1}$. A detailed derivation can be found in “Appendix 1”.

The gradient can be decomposed in attraction and repelling forces between points. Thus the underlying problem of ct-SNE, just like many other force-based embedding methods, is related to the classic *n*-body problem in physics,^3^ which has also been studied in the recent machine learning literature (Gray and Moore 2001; Ram et al. 2009). The general goal of the *n*-body problem is to find a constellation of *n* objects such that equilibrium is achieved according to a certain measure (e.g., forces, energy). In the problem setting of ct-SNE, both the pairwise distances between points and the label information affect the attraction and repelling forces. Particularly, the label information strengthens the repelling force between two points if they have the same label and weakens the repelling force if two points have different labels (when $\alpha ^{\prime}> 1> \beta ^{\prime} > 0$). This is desirable because we do not need to reflect the known label information in the resulting embeddings.

Evaluating the gradient has complexity ${\mathcal {O}}(n^2)$, which makes the computation (both time and memory cost) infeasible when *n* is large (e.g., $n> 100k$). As an approximation of the gradient computation, we adapt the tree-based approximation strategy described by van der Maaten (2014). To efficiently model the proximity in high-dimensional space (Eq. 1) we use a vantage-point tree-based algorithm (which exploits the fast diminishing property of the Gaussian distribution). To approximate the low-dimensional proximity (Eq. 3) we modify the Barnes-Hut algorithm to incorporate the label information. The basic idea of the Barnes-Hut algorithm is to organize the points in the embedding space using a kd-tree. Each node of the tree corresponds to a cell (dissection) in the embedding space. If a target point ${\varvec{y}}_i$ is far away from all the points in a given cell, then the interaction between the target point and the points within the cell can be summarized by the interaction between ${\varvec{y}}_i$ and the cell’s center of mass ${\varvec{y}}_{\text {cell}}$ that is computed while constructing the kd-tree. More specifically, the summarization happens when $r_{\text {cell}}/\Vert {\varvec{y}}_i - {\varvec{y}}_{\text {cell}}\Vert ^2 < \theta$, where $r_{\text {cell}}$ is the radius of the cell, while $\theta$ controls the strength of summarization, i.e. the approximation strength. The summarized repelling force in t-SNE reads $F_{\text {rep}} = -n_{\text {cell}}q^2_{i,\text {cell}}Z({\varvec{y}}_i - {\varvec{y}}_{cell})$, where $n_{\text {cell}}$ is the number of data points in that cell.

For ct-SNE, we have to overcome an additional complication though: we also need to summarize the label information for the points in a cell when the summarization happens. This can be done by maintaining a histogram in each cell, and counting the numbers of data points with different labels that fall into that cell. Then the repelling force of a target point ${\varvec{y}}_i$ can be weighted proportional to the number of points that have equal/different labels within the cell. Namely:

$$\begin{aligned} F^{\text {approx.}}_{\text {rep}} =-\frac{\alpha ^{\prime}n_{{\text {cell}},l = l_i} + \beta ^{\prime}(n_{\text {cell}} - n_{{\text {cell}},l = l_i} )}{O} q^2_{i,{\text {cell}}}Z({\varvec{y}}_i - {\varvec{y}}_{cell}), \end{aligned}$$

where $n_{{\text {cell}},l = l_i}$ is the number of data points in a cell that have the same label as point ${\varvec{y}}_i$.

As both tree-based approximation schemes have complexity ${\mathcal {O}}\left( n\log n\right)$, counting the label will add an extra multiplicative constant *L*, equal to the number of label values in the prior information. Thus the final complexity of approximated ct-SNE is ${\mathcal {O}}\left( L\cdot n\log n \right)$. We summarize ct-SNE in Algorithm 1.
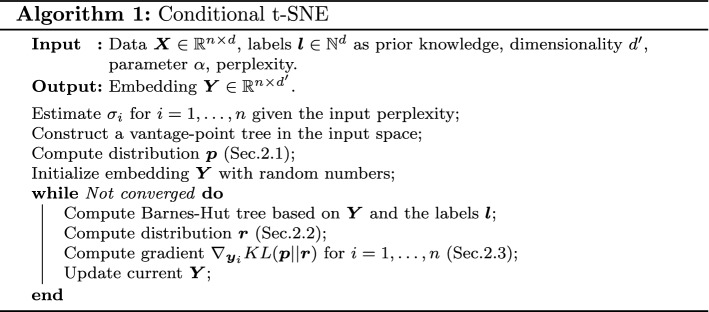


### Conditioning for other DR methods

The idea of discounting known factors from low-dimensional representations can be generalized to other *n*-body problem based DR methods. Oftentimes, the gradient of the *n*-body problem based methods can be viewed as a summation of attraction forces and repelling forces. Removing a known factor thus amounts to re-weighting the attracting and repelling forces such that points that have the same label repel each other and points with different labels attract each other.

For example, LargeVis (Tang et al. 2016) differs from t-SNE by modeling input space proximity using a random k-NN graph. Thus we can use the same conditioning idea in LargeVis as in ct-SNE to remove known factors. However, for Uniform Manifold Approximation and Projection (UMAP) (McInnes and Healy 2018), conditioning is not readily applicable. In contrast to t-SNE, UMAP uses fuzzy sets to model the proximity in both input space and embedding space. Then the cross entropy between two fuzzy sets serves as loss function to compare the modeled proximity between input space and the embedding space. In the UMAP setting, it is not straightforward to condition the lower dimensional proximity model on the prior. But we can still directly re-weight the repelling forces: for data points with the same label, the pushing effect is strengthened by $\alpha$; for samples with different labels, the pushing effect is weakened by multiplying with $\beta$, assuming $\alpha> 1> \beta > 0$. However, without proper conditioning, parameter $\alpha$ and $\beta$ lose their probabilistic interpretation and their one-to-one correspondence, thus both $\alpha$ and $\beta$ need to be set.

## Experiments

We conducted experiments to investigate four questions: **Q1** Does ct-SNE work as expected in finding complementary structure? **Q2** How should the parameter $\alpha$ be chosen? **Q3** Could ct-SNE’s goal also be achieved by using (a combination of) other methods? **Q4** How well does ct-SNE scale? In this section we mainly focus on 2D visualizations. Although the method works in higher dimensions, three dimensional embeddings would pose additional challenges for visualization in this paper and interpretation by the reader. We encourage the users to use our open source implementation to explore the ct-SNE embeddings in higher dimensions.

Sections 3.2–3.4 contain four case studies addressing **Q1**. The experiments addressing **Q2** are discussed in Sect. 3.6. Section 3.7 contains parameter sensitivity analysis that addressing **Q3**. Research question **Q4** is studied in Sect. 3.8.

We first introduce the data and the experimental setup.

### Data and experimental settings

The first dataset is a **Synthetic dataset.** consisting of 1000 ten-dimensional data points, used also in the example in Sect. 1. The first four dimensions are generated by placing each of the points into one of five clusters and adding unit variance Gaussian noise on each dimension. For the next two dimensions, points are independently placed into four clusters located in this 2D subspace, again adding unit-variance Gaussian noise. The remaining four dimensions are just zero-mean unit-variance Gaussian noise. As the data can be clustered over unrelated subspaces, there exists no embedding that shows both clusterings as coherent clusterings concurrently.

The second dataset is a **UCI Adult dataset.** We sampled 1000 data points from the UCI Adult dataset (Dheeru and Karra Taniskidou 2017) with six attributes: the three numeric attributes *age*, *education level*, and *work hours per week*, and the three binary attributes *ethnicity* (white/other), *gender*, and *income (> 50k)*.

The third dataset is a **DBLP dataset.** We extracted all papers from 20 venues^4^ in four areas (ML/DM/DB/IR) of computer science from the DBLP citation network dataset (Tang et al. 2008). We sampled half of the papers and constructed a network (122,962 nodes^5^) based on paper-author, paper-topic, paper-venue relations. Finally, we embedded the network into a 64 dimensional euclidean space using node2vec (Grover and Leskovec 2016) with walk length 80, window size 10. In our experiment, both *p* and *q* are set to 1.^6^ Under this setting, node2vec is equivalent to DeepWalk (Perozzi et al. 2014).

The forth dataset is a **Facebook dataset.** consisting of 128-dimensional embedding of a de-identified random sample of 500*k* Facebook users in the US. This embedding is generated purely based on the list of pages and groups that the users follow, as part of an effort to improve the quality of several recommendation systems at Facebook.

To study **Q1**, both qualitative and quantitative experiments were performed on the synthetic, UCI Adult, and DBLP datasets. On the Facebook dataset we only conducted a qualitative evaluation (given the lack of ground truth).

#### Qualitative experiments setup

We evaluated the effectiveness of ct-SNE qualitatively through visualizations. More specifically, we compare the t-SNE visualization of a dataset with the ct-SNE visualization that has taken into account certain prior information that is visually identifiable from the t-SNE embedding. Thus by inspecting the presence of the prior information in the ct-SNE embedding and comparing to the t-SNE embedding, we can evaluate whether the prior information is removed. Conversely, we test whether information present in the ct-SNE embedding could have been identified from the t-SNE embedding to verify whether it contains complementary information.

To select the prior information, we visualized the t-SNE embedding and manually selected points that appear clustered. Then we performed a *feature ranking* procedure to identify the features that separate the selected points from the rest. This was done by fitting a linear classifier (logistic regression) on the selected cluster against all other data points. By inspecting the classifier weights, we identified the features with largest contribution in aforementioned classification task. Repeating this *feature ranking* procedure for other clusters, we aimed to find a feature that correlates with the majority of the clusters in the t-SNE visualization. This feature was then treated as prior information and provided as input to ct-SNE. In the reported experiments, the most prominent feature was always categorical, so all points with the same value were treated as being in a cluster to define the prior. We used exact ct-SNE on the Synthetic and approximated ct-SNE ($\theta =0.5$) on the Facebook dataset.

We also evaluated whether ct-SNE can provide deeper insights, by iteratively embedding data, each time applying cluster selection and feature ranking.

#### Quantitative experiments setup

In this experiment, we quantify the presence of certain prior information in a ct-SNE embedding that uses the same prior information as input. For example, the presence of label information in an embedding can be measured by considering the homogeneity of those labels in the embedding, i.e., points that are close to each other in the embedding often have the same label. To quantify such homogeneity, we developed a measure termed *normalized Laplacian score*: Given an embedding ${\varvec{Y}}$ and parameter *k*, we denote ${\varvec{A}}_k$ as the adjacency matrix of the k-Nearest Neighbor (k-NN) graph computed from the embedding. The Laplacian matrix of the k-NN graph has the form ${\varvec{L}}_k = {\varvec{A}}_k - {\varvec{D}}_k$ where ${\varvec{D}}_k = {\text {diag}}({\text {sum}}({\varvec{A}}_k, 1))$. We further normalize the Laplacian matrix (${\varvec{D}}_k^{-1/2}{\varvec{L}}_k{\varvec{D}}_k^{-1/2}$) to obtain a score that is insensitive to node degrees. Given a label vector ${\varvec{f}}$ with *L* values where each label *l* has $n_l$ points, and denoting the one-hot encoding for each label *l* as ${\varvec{f}}_l$, the normalized Laplacian score is:

9 $$\begin{aligned} \sum _{l \in [0..L]} \frac{n_l}{n} \frac{{\varvec{f}}_l^{\prime}{\varvec{D}}_k^{-1/2}{\varvec{L}}_k{\varvec{D}}_k^{-1/2}{\varvec{f}}_l}{{\varvec{f}}_l^{\prime}{\varvec{f}}_l}. \end{aligned}$$

This score has range [0, 1].^7^ Roughly speaking, the normalized Lapacian score is a measure for how often the labels of nodes connected in the k-NN graph differ from each other. If a label is locally coherent (homogeneous) in an embedding, the feature difference in k-NN graph neighborhoods is small, which results in a small Laplacian score. Conversely, a heterogeneous label over the k-NN graph would have a large Laplacian score. Thus, if an embedding has large Laplacian score for the labels used as prior information, ct-SNE effectively removed certain prior information from the embedding. In “Appendix 3” we walk through an example to demonstrate the usage of the normalized Laplacian score.

For comparison, we also plotted the normalized Laplacian score for a randomly permuted label assignment on the k-NN graph. As we will see in the experiments, the normalized Laplacian scores for these randomized label vectors are often substantially smaller than the theoretical upper bound of 1, although they are still larger than almost all other scores obtained by the t-SNE/ct-SNE embeddings in our case studies. Thus, the normalized Laplacian score for a randomized labeling elucidates in a simple manner what Laplacian score ct-SNE could achieve if it were able to entirely remove any dependency between proximity of the data points and their labels, for the given label distribution. As such it provides an insightful benchmark, helping one to understand the significance of any improvement in normalized Laplacian score achieved by ct-SNE as compared to t-SNE.

### Case study: synthetic dataset

#### Qualitative experiment

The t-SNE visualization of the synthetic dataset shows five large clusters (Fig. 1a). Feature ranking (Sect. 3.1 ’Qualitative experiments setup’) shows these clusters correspond to the clustering in dimensions 1–4 of the data. Taking the cluster labels in dimensions 1–4 (${\varvec{f}}_{1{-}4}$) as prior, ct-SNE gives a different visualization (Fig. 1b). The feature ranking further shows this ct-SNE embedding indeed reveals the clusters in the dimension 5–6 of the data. We combined the labels ${\varvec{f}}_{1{-}4}$ and ${\varvec{f}}_{5{-}6}$ by assigning a new label to each combinations of the labels, denoted as ${\varvec{f}}_{1{-}6}$. ct-SNE with ${\varvec{f}}_{1{-}6}$ yields an embedding based only on the remaining noise (Fig. 1c). Detailed feature ranking results and cluster statistics are reported in “Embeddings of synthetic dataset” section of “Appendix”.

#### Quantitative experiment

**Fig. 2:**
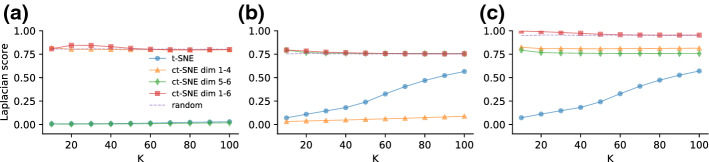
The homogeneity of cluster labels in t-SNE and several ct-SNE embeddings of the synthetic dataset for *k* (a parameter of the Laplacian score) ranging from 10 to 100, for the three label sets: **a** ${\varvec{f}}_{1{-}4}$, **b** ${\varvec{f}}_{5{-}6}$, and **c** ${\varvec{f}}_{1{-}6}$. Colored lines give the scores for different embeddings: t-SNE (blue), ct-SNE with prior ${\varvec{f}}_{1{-}4}$ (orange), ct-SNE with prior ${\varvec{f}}_{5{-}6}$ (green), ct-SNE with prior ${\varvec{f}}_{1{-}6}$ (red). Laplacian scores obtained by randomly permuting the labels are plotted in dashed lines in all three plots (Color figure online)

We computed the normalized Laplacian scores (see Eq. 9) for the t-SNE and ct-SNE embeddings. Subfigures in Fig. 2a–c give the Laplacian score for three label sets: ${\varvec{f}}_{1{-}4}$, ${\varvec{f}}_{5{-}6}$, and ${\varvec{f}}_{1{-}6}$. Figure 2a shows that labels ${\varvec{f}}_{1{-}4}$ are less homogeneous (higher Laplacian score) in the ct-SNE embeddings with prior ${\varvec{f}}_{1{-}4}$ and ${\varvec{f}}_{1{-}6}$ than in the t-SNE embedding, indicating that ct-SNE effectively discounted the prior from the embeddings. Both the t-SNE embedding and ct-SNE with prior ${\varvec{f}}_{5{-}6}$ pick up the clustering in ${\varvec{f}}_{1{-}4}$, as indicated by the low Laplacian score. Similarly, Fig. 2b, c show that ct-SNE removes the prior information effectively for labels ${\varvec{f}}_{5{-}6}$ and ${\varvec{f}}_{1{-}6}$, respectively, given the associated priors.

### Case study: UCI Adult dataset

#### Qualitative experiment

**Fig. 3:**
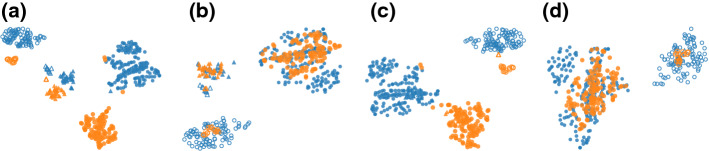
Visualization of 2-d embeddings of the UCI Adult dataset. Points are visually encoded according to their attributes. *gender*: *female* (orange color), *male* (blue color); *ethnicity*: *white* (circle), *other* (triangle); *income (> 50k)*: *true* (unfilled marker), *false* (filled marker). **a** t-SNE embedding shows clusters that are grouped according to the combinations of all three attributes. **b** With attribute *gender* as prior, ct-SNE embedding shows four clusters each has a mixture of points with different genders, indicating the *gender* information is removed. **c** With attribute *ethnicity* as prior, ct-SNE embedding also shows four clusters but each has a mixture of points with different ethnicities. **d** Incorporating the combination of attributes *gender* and *ethnicity* as prior, the resulted ct-SNE embedding shows two clusters that are correlated with the remaining attribute: *income (> 50k) * (Color figure online)

Figure 3a shows t-SNE gives an embedding that consists of clusters grouped according to combinations of three attributes: *gender*, *ethnicity* and *income (>50k)*. By incorporating the attribute *gender* as prior, the ct-SNE embedding (Fig. 3b) contains clusters with a mixture of *male* and *female* points, indicating the *gender* information is removed. Instead, by incorporating the attribute *ethnicity* the ct-SNE embedding (Fig. 3c) contains clusters with a mixture of ethnicities. Finally, incorporating the combination of attributes *gender* and *ethnicity* as prior, the ct-SNE embedding contains data points grouped according to *income* (Fig. 3d). Detailed feature ranking results and cluster statistics are reported in “Embeddings of UCI Adult dataset” section of “Appendix”.

#### Quantitative experiment

**Fig. 4:**
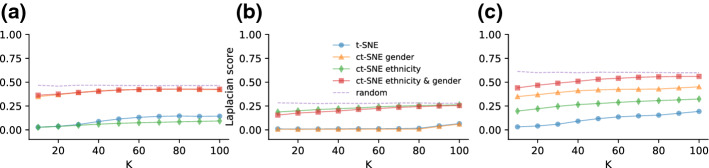
The homogeneity of cluster labels in t-SNE and several ct-SNE embeddings of the UCI Adult dataset for *k* (a parameter of the Laplacian score) ranging from 10 to 100 with step size 10. Colored lines correspond to scores for different embeddings: t-SNE (blue), ct-SNE with prior *gender* (orange), ct-SNE with prior *ethnicity* (green), and ct-SNE with prior *ethnicity & gender* (red). Subfigures give homogeneity scores for various labels: **a** *gender*, **b** *ethnicity*, **c** *gender* & *ethnicity*. **a** The attribute *gender* has lower homogeneity (high Laplacian score) in the ct-SNE embedding with *gender* or *ethnicity & gender* as prior than in t-SNE embedding and ct-SNE embedding with *ethnicity* as prior. **b** The attribute *ethnicity* has lower homogeneity in the ct-SNE embedding with *ethnicity* or *ethnicity & gender* as priors than in the t-SNE embedding and ct-SNE with *gender* as prior) embeddings. **c** The attribute *ethnicity & gender* has high homogeneity in the t-SNE embedding only. Laplacian scores obtained by randomly permuting the labels are plotted in dashed lines in all three plots (Color figure online)

We analyzed the homogeneities (Laplacian scores) of attributes *gender*, *ethnicity* and *income (>50k)* measured on both t-SNE and ct-SNE embeddings. Figure 4a shows ct-SNE with prior *gender* removes the *gender* factor from the resulted embedding, while ct-SNE with prior *ethnicity* makes the *gender* factor in the resulted embedding clearer. Similarly, Fig. 4b, c show ct-SNE removes the prior information effectively for labels *ethnicity* and *ethnicity&gender* respectively, given the associated priors.

### Case study: DBLP dataset

#### Qualitative experiment

**Fig. 5:**
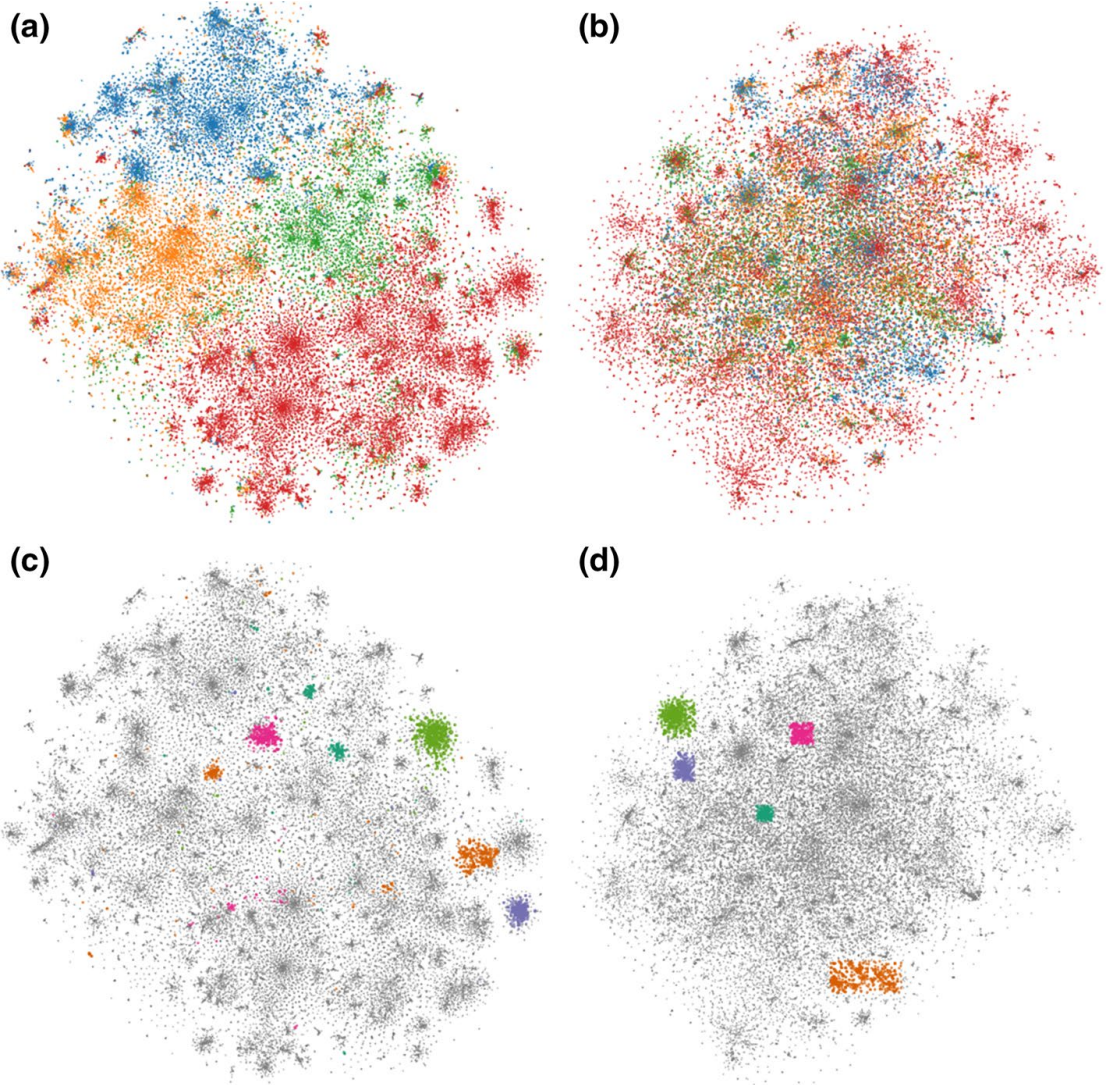
Visualization of 2-d embeddings of the DBLP dataset. Left column: t-SNE embedding, right column: ct-SNE embedding with area as prior. The rows contains different cluster markings. **a** t-SNE embedding shows a clustering according to four areas in computer science (red—machine learning, green—data mining, blue—data base, orange—information retrieval). **b** ct-SNE embedding shows a different clustering, with area information removed. **d** Newly emerged visual clusters (magenta—topic ‘privacy’, dark green—topic ‘data stream’, orange—topic ‘computer vision’) in ct-SNE embedding spread over in the t-SNE embedding (**c**), corresponding to users interested in horse riding. **d** Clusters (grass green—topic ‘clustering’, purple—topic ‘active leraning’) stood-out in the ct-SNE embedding also exists in the t-SNE embedding (**c**). These are a few out of many clusters that we found to exhibit a much more informative, interest-centric structure than the t-SNE projection (Color figure online)

Applying t-SNE on the DBLP dataset gives a visualization with many visual clusters (Fig. 5a). Feature ranking for classification of the selected clusters shows the topics that contribute the most to the visualization. Moreover, we used mpld3^8^ (an interactive visualization library) to inspect (i.e., hovering over data points and check tooltips) the metadata of t-SNE plot. Upon inspection, the visualization appears to be globally divided according the four areas. This is further confirmed by coloring the data points according to the four areas: most of the clusters are indeed quite homogeneous with respect areas.

Knowing from the t-SNE visualization the papers are indeed divided according to areas, the area structure in the visualization is not very informative anymore. Thus we can encode the area as prior for ct-SNE so that other interesting structures can emerge. Using the same color scheme, ct-SNE shows a visualization that has many clusters with mixed colors (Fig. 5b). This indicates the area information is mostly removed in the ct-SNE embedding. This is further confirmed by selecting clusters in ct-SNE embedding (Fig. 5d) and highlight the same set of points in the t-SNE embedding (Fig. 5c). The clusters highlighted in the ct-SNE visualization often consists of clusters (topics) from different areas (i.e., t-SNE clusters with different colors) that spread over the t-SNE visualization. Indeed, feature ranking indicates that papers in the selected ct-SNE cluster have similar topics in e.g., ‘privacy’, ‘data steam’, ‘computer vision’. Finally, we noticed that some clusters in ct-SNE (Fig. 5d) embedding also exist in the t-SNE embedding (Fig. 5c). Using feature ranking as above we found these clusters are not homogeneous in terms of area of study, but in terms of topics (e.g., ‘clustering’, ‘active learning’), indicating a tightly connected research community behind the topic. Thus, by removing the irrelevant area structure using ct-SNE, clusters that persists in both visualizations become more salient and easier to observe. Frequent topics in the clusters are reported in “Embeddings of DBLP dataset” section of “Appendix”.

#### Quantitative experiment

**Fig. 6:**
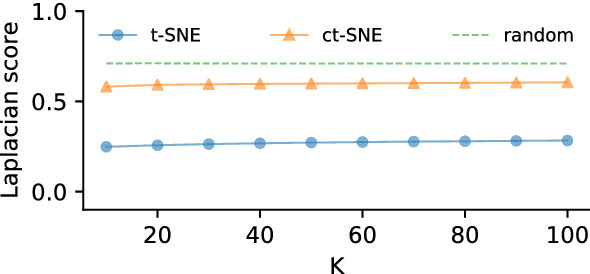
The homogeneity of cluster labels in t-SNE and several ct-SNE embeddings of the DBLP dataset for *k* (a parameter of the Laplacian score) ranging from 10 to 100 with step size 10. Colored lines correspond to scores for different embeddings: t-SNE (blue), ct-SNE with prior *area* (orange). The attribute *area* has lower homogeneity (high Laplacian score) in the ct-SNE embedding with *area* as prior than in t-SNE embedding. Laplacian scores obtained by randomly permuting the labels are plotted in dashed line (Color figure online)

We analyzed the homogeneities (Laplacian scores) of paper area structure measured on both t-SNE and ct-SNE embeddings. Figure 6 shows ct-SNE with prior *area* removes the *area* factor from the resulted embedding.

#### Remark

Note that the maximal value of the normalized Laplacian scores will be affected by the imbalance of the labels in the prior. More specifically, if the majority of the data points have the same label value (e.g., “ethnicity” = “white” in UCI Adult dataset), then the neighbors of each data point in the k-NN graph would be more likely to have the same label. As a result, normalized Laplacian scores would be smaller for larger imbalance of the labels. This explains the observation in the UCI Adult case (Fig. 4) where the effect of ct-SNE removing known factors as measured by the normalized Laplacian score is not as large as in the other cases where the label values are more balanced (Figs. 2, 6). The random benchmark introduced in Sect. 3.1 empirically achieves normalized Laplacian scores smaller than the theoretical upper bound but larger than almost all other scores obtained by the t-SNE/ct-SNE embeddings in our case studies. This benchmark thus allows one to make more sensible comparisons between different methods.

### Case study: Facebook dataset

#### Qualitative experiment

**Fig. 7:**
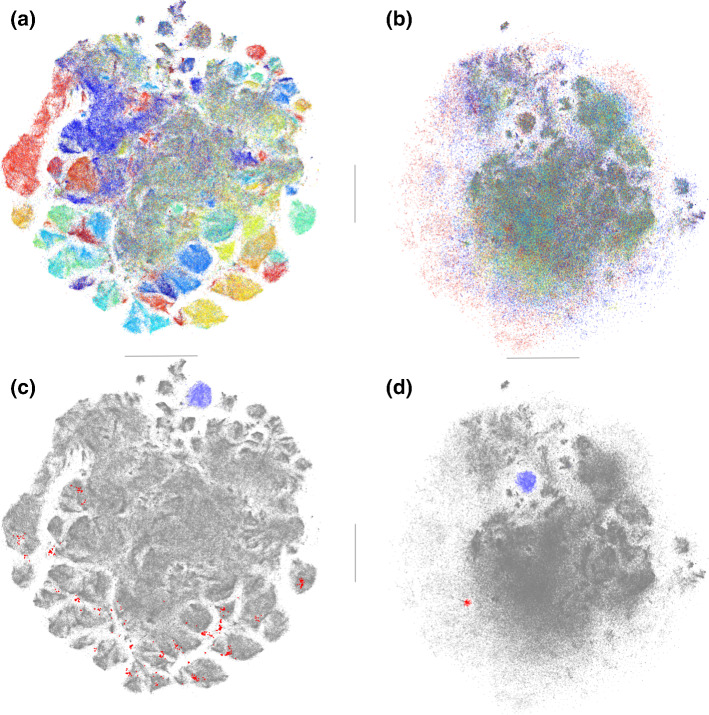
Visualization of 2-d embeddings of the Facebook dataset. Left column: t-SNE embedding, right column: ct-SNE embedding with region as prior. The two rows show identical embeddings but with different cluster markings (colors). See Sect. 3.5 for further info (Color figure online)

Applying t-SNE on the Facebook dataset gives a visualization with many visually salient clusters (Fig. 7a). Computing the feature ranking for classification of selected clusters shows that the geography (i.e., the states) contributes to the embedding the most. This is confirmed by coloring the data points according to the geographical region in the visualization as shown in Fig. 7a: most of the clusters are indeed homogeneous in geographic location.

To understand the use of an embedding like this in a downstream recommendation system, consider that an analyst typically wants to know what type of user interests the embedding is capturing. The fact that there are regional clusters is not very informative. To obtain a more useful embedding, we can encode the region as prior for ct-SNE so that other interesting structures can emerge.

Using the same coloring scheme, ct-SNE shows a cluster with large mass that consists of users from different states (Fig. 7b). There are also a few small clusters with mixed color scattered on the periphery of the visualization. The visualization indicates that geographical information is almost absent in the ct-SNE embedding. This is further confirmed by selecting clusters (highlighted in red color) in ct-SNE embedding (Fig. 7d) and highlighting the same set of points in the t-SNE embedding (Fig. 7c). The cluster highlighted in the ct-SNE embedding spreads over the t-SNE embedding, indicating these users are not geographically similar. Indeed, the feature ranking procedure indicates that the selected group of users (Fig. 7d) share an interest in horse riding: they tend to follow several pages related to that topic. Interestingly, we noticed that some of the clusters in the ct-SNE embedding are also clustered in the t-SNE embedding. These clusters are indeed heterogeneous in terms of the geographical regions. For example, the cluster highlighted in blue in the ct-SNE embedding (Fig. 7d) also exists in the t-SNE embedding (Fig. 7c). Using feature ranking as above we found that these clusters are homogeneous terms of users’ interest in Indian culture. While these clusters can thus also be seen in the t-SNE embedding, ct-SNE removes the region cluster structure, such that those other clusters become more salient.

### Parameters sensitivity

To understand the effect of the parameter $\alpha ^{\prime}$ (or equivalently, $\beta ^{\prime}$) on ct-SNE embeddings (**Q3**), we study ct-SNE embeddings on the synthetic dataset with the prior fixed to be the cluster labels in dimensions 1–4. First, we try to understand the relation between the ct-SNE objective and the parameter $\alpha ^{\prime}$ (or equivalently, $\beta ^{\prime}$). We evaluated the ct-SNE objective (Eq. 5) on the ct-SNE embeddings obtained by ranging $\beta ^{\prime}$ (and $\alpha ^{\prime}$ correspondingly) from 0.01 (strong prior removal effect) to 1.0 (no prior remove effect, equivalent to t-SNE) with step size 0.1. We also evaluated the t-SNE objective (first term in Eq. 5) and the second term in Eq. 5 (the only term that depends on the prior, subsequently referred to as the *prior term*) for the ct-SNE embeddings associated with various $\beta ^{\prime}$s.

**Fig. 8:**
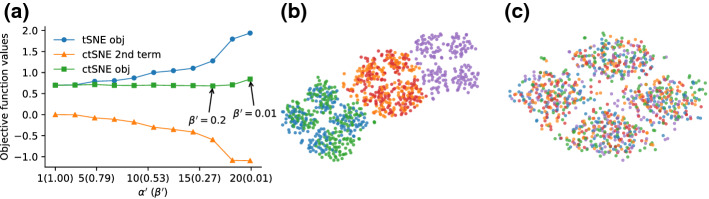
Visualizing the effect of different $\beta ^{\prime}$s ($\alpha ^{\prime}$s) have on the ct-SNE embeddings. The embeddings are computed on the synthetic dataset with the prior information to be the cluster labels in dimensions 1–4. **a** The values of ct-SNE objective (green), t-SNE objective (blue), and ct-SNE prior term (orange) against different $\beta ^{\prime}$s. ct-SNE achieves smaller KL-divergence than t-SNE. **b** ct-SNE embedding with $\beta ^{\prime} = 0.2$ has smallest KL-divergences but is not the best visualization. **c** ct-SNE embedding with $\beta ^{\prime}=0.01$ gives a better visualization (Color figure online)

Figure 8a visualizes the values of the ct-SNE objective, t-SNE objective, and ct-SNE prior term against different $\beta ^{\prime}$s. Observe that by using a prior, the ct-SNE embedding achieves a better approximation to the higher dimensional data. That is, ct-SNE achieves a lower KL-divergence (lowest at $\beta ^{\prime} = 0.2$) than t-SNE does ($\beta ^{\prime} = 1$). This is because the prior term in the ct-SNE objective can be negative. Although the t-SNE objective increases when $\beta ^{\prime}$ decreases, it is compensated by the negative value contributed by the prior term. Indeed, by factoring out certain prior from the lower dimensional embedding, the necessity of the embedding to represent the prior is alleviated, enabling ct-SNE to have more freedom to approximate the high-dimensional proximities.

Interestingly, we observe that the embedding with smallest KL-divergence does not necessarily give better visualization (e.g., clear separation of the clusters). We visualize the ct-SNE embedding that achieves smallest KL-divergence ($\beta ^{\prime}=0.2$, Fig. 8b) and compare it with the ct-SNE embedding that has strongest prior removal effect but larger KL-divergence ($\beta ^{\prime}=0.01$, Fig. 8c). Although the embedding with stronger prior removal effect has larger objective value, it gives a clearer clustering than in the embedding with smaller KL-divergence ($\beta ^{\prime}=0.2$). As a result, the clusters in dimensions 5–6 are easier to identify. Hence, we propose as rule of thumb when using ct-SNE for visualization to use small $\beta ^{\prime}$ (e.g., $\beta ^{\prime}=0.01$).

### Baseline comparisons

In this section, we compare ct-SNE with two non-trivial baselines. The basic idea is to first remove the known factor from the dataset, and perform t-SNE to produce lower dimensional representations. Here we use a non-linear and a linear method to remove the known factors: adversarial auto-encoder (AAE) and canonical correlation analysis (CCA).

#### Baseline: AAE and t-SNE

Adversarial auto-encoder (AAE) (Makhzani et al. 2015) can be used to learn a latent representation that prevents the discriminator from predicting certain attributes (Madras et al. 2018). In order to remove prior information from the low-dimensional representation of a dataset using AAE, we can configure the discriminator to predict the prior attributes, and using the auto-encoder to adversarially remove the prior from the latent representation of the dataset.

**Fig. 9:**
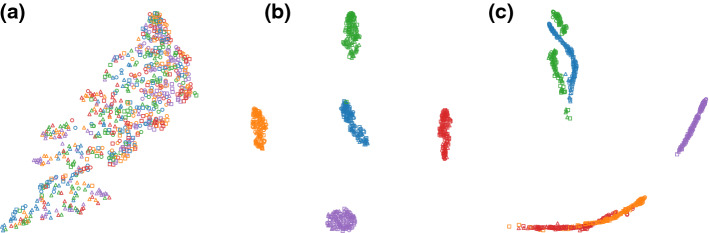
Visualization of 2-d embeddings obtained by applying the AAE based approach on the synthetic dataset. The data points are colored according to the cluster label in dimensions 1–4. The data points are also plotted using different markers based on the cluster labels in dimensions 5–6. **a** The AAE based approach successfully removed the clustering information in dimensions 1–4, but failed to reveal the clusters in dimensions 5–6 (**b**) AAE successfully removed the clustering information in dimensions 5–6 and also reveals the clusters in dimensions 1–4 (**c**) AAE failed to remove the clustering information in dimensions 1–6 (Color figure online)

We adopt the AAE configuration described by Edwards and Storkey (2015). AAE is in general difficult to tune: it has 8 hyperparameters (4 network structure parameters, 2 weights in the objective, and 2 learning rates) and a few design choices about the network architecture (e.g., the number of layers in each subnetwork and activation functions). We tried different parameter settings and managed to remove the clustering label information in dimensions 1–4 (Fig. 9a) and 5–6 (Fig. 9b) from the data. In Fig. 9a, the AAE approach manages to remove the prior information, but it fails to pick up the complementary structure in the data (clusters in dimensions 5–6). It also fails to remove the prior information (cluster labels in dimension 1–6) in Fig. 9c. Comparing to this baseline, ct-SNE practically has only one parameter ($\beta ^{\prime}$) to tune, which often can be set to a small positive number (e.g., 0.01).

#### Baseline: CCA and t-SNE

Canonical correlation analysis (Hotelling 1936) aims to find a linear transformation for two random variables such that the correlation between transformed variables is maximized. To remove the prior information from data using CCA, one approach is to first find the (at most) $d-2$ subspace (*d* is the dimensionality of the data) in which the transformed data and the prior information (one hot encoding of the labels) have the largest correlation. Then the data is whitened by projecting it onto the null space (at least 2-d) of the subspace found in the first step. By doing so, the whitened data is less correlated to the known factor.

Another variant of the CCA-based approach is directly projecting the data onto the 2-dimensional subspace found by CCA in which the transformed data and labels has smallest correlation. To be consistent, we also apply t-SNE to the transformed data.

**Fig. 10:**
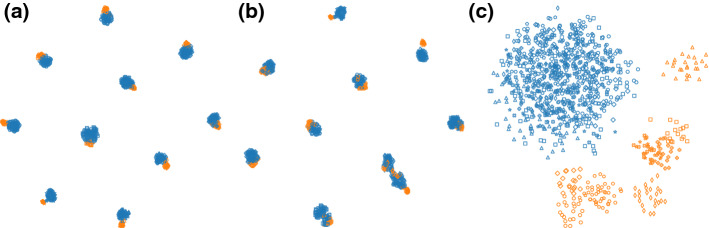
Visualization of 2-d embeddings obtained by applying CCA-based approaches and ct-SNE on a synthetic 5 dimensional dataset. **a** Projecting data onto the null space of CCA top components and then apply t-SNE gives an embedding that picks up the 10 large clusters (plotted with different markers) but failed to pick up the structure of two small clusters (colored differently) within each large cluster. **b** Projecting the data onto CCA components with least correlation and then apply t-SNE also fails to pick up the two-cluster structure within the large clusters. **c** ct-SNE removes the 10 cluster information in the embedding and shows clearly the two cluster structure within each larger cluster

Our experimental results show the CCA-based approaches can easily remove label information that is orthogonal to other attributes in the data. For example, in the UCI Adult dataset, the gender information is orthogonal to the ethnicity and income, which can be easily removed using the CCA approach. However, the CCA-based approach performs poorly when the known factor is correlated with other attributes. Moreover, the CCA-based approaches also have the limitation that the number of the projection vectors is upper-bounded by the dimensionality of the data. If the number of unique values of an attribute exceeds the dimensionality of the data, the CCA projection would not be able to remove the label info entirely from the data. To illustrate our points, we synthesized a 5-dimensional dataset with 1000 data points. The data points are grouped into 10 clusters each corresponding to a multi-variate Gaussian with random location and small variance. Additionally, each cluster is separated into two small clusters (one contains $20\%$ points of the cluster, and another includes the rest) along one randomly chosen axis. Figure 10a, b shows both the CCA approaches pick up only the 10 large clusters (differentiated using marker shape) but failed to pick up the structure of two small clusters (plotted in different colors) within each large cluster. On the other hand, ct-SNE removes the 10 cluster information in the embedding and shows each large cluster can be further separated in to two smaller clusters.

Thus, the CCA-based baselines perform poorly when the known factor is correlated with other attributes. Moreover, the number of the projection vectors in CCA-based baselines is upper-bounded by the dimensionality of the data. Meanwhile, ct-SNE does not have these limitations.

### Runtime

We measure the runtime of the exact ct-SNE and the approximated version ($\theta =0.5$) on a PC with a quad-core 2.3 GHz Inter Core i5 and a 2133 MHz LPDDR3 RAM. By default, the maximum number of iterations of ct-SNE gradient update is 1000. For larger datasets and prior attributes that have many values, more iterations are required to achieve a convergence. For example, the synthetic dataset (1000 samples and 10 dimensions) requires fewer than 1000 iterations to converge while the Facebook dataset (500,000 examples and 128 dimensions) requires 3000 iterations to converge. Table  1 shows that approximated ct-SNE is efficient and applicable to large data with high dimensionality, while exact ct-SNE is not.

**Table 1:** Average runtime (in seconds) of exact and approximated ct-SNE in computing one gradient update step

Name	Size	Dim.	Exact	Apprx. ($\theta = 0.5$)
Synthetic	1000	10	0.06	0.01
UCI Adult	1000	6	0.07	0.01
DBLP	43,346	64	503.97	0.45
Synthetic	500,000	128	100,278	9.1

To measure the runtime of ct-SNE on a dataset with similar size as the Facebook dataset, we scaled the Synthetic dataset up to 500,000 data points with 128 dimensions

## Related work

Many dimensionality reduction methods have been proposed in the literature. Arguably, *n*-body problem based methods^9^ such as MDS (Torgerson 1952), Isomap (Tenenbaum et al. 2000), t-SNE (van der Maaten and Hinton 2008), LargeVis (Tang et al. 2016), and UMAP (McInnes and Healy 2018) appear to be the most popular ones. These methods typically have three components: (1) a proximity measure in the input space, (2) a proximity measure in the embedding space, (3) a loss function comparing the proximity between data points in the embedding space with the proximity in the input space. When minimizing the loss over the embedding space, the data points (i.e., the *n* bodies) have pairwise interactions and the embedding of all points needs to be updated simultaneously. Since the optimization problem is not convex, local minima are typically accepted as output. ct-SNE belongs to this class of DR methods. It accepts both high-dimensional data and priors about the data as inputs, and searches for low-dimensional embeddings while discounting structure in the input data specified as prior knowledge. Closely related, in the multi-maps t-SNE work (van der Maaten and Hinton 2012) factors that are mutually exclusive are captured by multiple t-SNE embeddings at once. Comparing to multi-map t-SNE, ct-SNE allows users to disentangle information in a targeted (subjective) manner, by specifying which information they would like to have factored out.

As a core component of ct-SNE is the prior information specified by the user, it can be considered an interactive DR method. Existing papers on *interactive* DR can be categorized into two groups. The first group aim to improve the explainability and computation efficiency of existing DR methods via novel visualizations and interactions. iPCA (Jeong et al. 2009) allows users to easily explore the PCA components and thus achieve better understanding of the linear projections of the data onto different PCA components. Cavallo and Demiralp (2018) helps the user to understand low-dimensional representations by applying perturbations to probe the connection between input attributed space and embedding space. Similarly, Faust et al. (2019) introduce a method based on perturbations to visualize the effect of a specific input attribute on the embedding, while Stahnke et al. (2016) introduce ‘probing’ as a means to understand the meaning of point set selections within the embedding. Steerable t-SNE (Pezzotti et al. 2017) aims to make t-SNE more scalable by quickly providing a sketch of an embedding which is then refined only upon the user’s interests.

The second group of interactive DR methods adjust the algorithms according to a users’ inputs. SICA (Kang et al. 2016) and SIDE (Puolamäki et al. 2018) explicitly model the user’s belief state and find linear projections that contrast to it. These two methods are linear DR methods thus cannot present non-linear structures in the low-dimensional representations. Work by Dıaz et al. (2014) allows users to define their own metric in the input space, after which the low-dimensional representation reflects the adjusted importance of the attributes. This method puts the burden on the user for direct manipulation of the input space metric. Many variants of existing DR methods have been introduced where user feedback entails editing of the embedding, and such manually embedded points are used as constraints to guide the dimensionality reduction (e.g., Alipanahi and Ghodsi 2011; Barshan et al. 2011; Paurat and Gärtner 2013). These methods contrast with ct-SNE in that the user feedback must be obeyed in the output embedding, while for ct-SNE the prior knowledge defined by the user guides what is irrelevant to the user.

## Conclusion

We introduce conditional t-SNE to efficiently discover *new* insights from high-dimensional data. ct-SNE finds the lower dimensional representation of the data in a non-linear fashion while removing the known factors. Extensive case studies on both synthetic and real-world datasets demonstrate that ct-SNE can effectively remove known factors from low-dimensional representations, allowing new structure to emerge and providing new insights to the analyst. A tree-based optimization method allows ct-SNE to scale to high dimensional data with hundreds of thousands of points.

As the future work, developing a more flexible way (e.g., continuous labels) of encoding the prior is certainly worth further investigation. Another interesting line of future work is to investigate the effect of different hyperparameter settings of ct-SNE. For example, if we set $0<\alpha ^{\prime}< 1< \beta ^{\prime}$, ct-SNE will instead of removing but finding low-dimensional representation that confirms the label similarity between the input data points. This is a desirable feature in confirmatory data analysis. Finally, generalizing the conditioning idea to other n-body problem-based methods is also worth exploring.

## Appendix 1: Detailed derivation of the gradient of the ct-SNE objective function

Here we derive in detail the gradient of the ct-SNE objective function. Denote the euclidean distance between points as $d_{ij} \triangleq \Vert {\varvec{y}}_i - {\varvec{y}}_j\Vert _2$. The derivative of $d_{ij}$ with respect to embedding ${\varvec{y}}_i$ reads:

$$\begin{aligned} \nabla _{{\varvec{y}}_i}d_{ij} = \frac{{\varvec{y}}_i - {\varvec{y}}_j}{d_{ij}}. \end{aligned}$$

Denote the cost (KL-divergence) by *C*:

$$\begin{aligned} C&= KL({\varvec{p}}\Vert {\varvec{r}}) \\&= C_1 + C_2 - \sum _{k\ne l: \delta _{kl}=1}p_{kl}\log (\alpha ^{\prime}) - \sum _{i\ne j: \delta =0}p_{kl}\log (\beta ^{\prime}), \end{aligned}$$

where

$$\begin{aligned} C_1 = KL({\varvec{p}}\Vert {\varvec{q}}), \end{aligned}$$

and

$$\begin{aligned} C_2 = \log \left( \alpha ^{\prime} \sum _{k\ne l: \delta _{kl} = 1}q_{kl} + \beta ^{\prime}\sum _{k\ne l: \delta _{kl} = 0}q_{kl}\right) . \end{aligned}$$

Following the derivation from tSNE paper, the derivative of $C_1$ with respect to ${\varvec{y}}_i$ reads:

$$\begin{aligned} \nabla _{{\varvec{y}}_i}C_1 = 4\sum _j(p_{ij} - q_{ij})(1 + \Vert {\varvec{y}}_i - {\varvec{y}}_j\Vert ^2)^{-1}({\varvec{y}}_i - {\varvec{y}}_j). \end{aligned}$$

To compute the derivative of $C_2$ with respect to ${\varvec{y}}_i$, we first have:

$$\begin{aligned} \nabla _{{\varvec{y}}_i}C_2 = 2\sum _j\frac{\partial C_2}{\partial d_{ij}} \cdot \frac{{\varvec{y}}_i-{\varvec{y}}_j}{d_{ij}}. \end{aligned}$$

Denote $O = \alpha ^{\prime} \sum _{i\ne j: \delta _{kl} = 1}q_{kl} + \beta ^{\prime}\sum _{i\ne j: \delta _{kl} = 0}q_{kl}$. The derivative of $C_2$ with respect to $d_{ij}$ is:

$$\begin{aligned} \frac{\partial C_2}{\partial d_{ij}}&= \frac{1}{O}\frac{\partial }{\partial d_{ij}}\left( \alpha ^{\prime} \sum _{k\ne l: \delta _{kl} = 1}q_{kl} + \beta ^{\prime}\sum _{k\ne l: \delta _{kl} = 0}q_{kl} \right) \\&= \frac{1}{O}\left( \alpha ^{\prime} \sum _{k\ne l: \delta _{kl} = 1}\frac{\partial q_{kl}}{\partial d_{ij}} + \beta ^{\prime}\sum _{k\ne l: \delta _{kl} = 0}\frac{\partial q_{kl}}{\partial d_{ij}} \right) \\&= \frac{1}{O}\Bigg (\alpha ^{\prime}\bigg (-2\delta _{ij}q_{ij}\left( 1+d_{ij}^2\right) ^{-1}d_{ij}\\&\quad +2\sum _{k\ne l:\delta _{kl}=1} q_{kl}q_{ij}\left( 1+d_{ij}^2\right) ^{-1}d_{ij}\bigg )\\&\quad+ \beta ^{\prime}\bigg (-2(1-\delta _{ij})q_{ij}(1+d_{ij}^2)^{-1}d_{ij} \\&\quad +2\sum _{k\ne l:\delta _{kl}=0} q_{kl}q_{ij}\left( 1+d_{ij}^2\right) ^{-1}d_{ij}\bigg )\Bigg )\\&= \frac{1}{O}\Bigg (2\alpha ^{\prime}\bigg (-\delta _{ij} +\sum _{k\ne l:\delta _{kl}=1} q_{kl}\bigg )q_{ij}\left( 1+d_{ij}^2\right) ^{-1}d_{ij}\\&\quad + 2\beta ^{\prime}\bigg (-(1-\delta _{ij}) +\sum _{k\ne l:\delta _{kl}= 0} q_{kl}\bigg )q_{ij}\left( 1+d_{ij}^2\right) ^{-1}d_{ij}\Bigg )\\&= 2\left( 1-\frac{\delta _{ij}\alpha ^{\prime} + (1-\delta _{ij})\beta ^{\prime}}{O}\right) \cdot q_{ij}\left( 1+d_{ij}^2\right) ^{-1} d_{ij}. \end{aligned}$$

Thus we have derivative of $C_2$ with respect to ${\varvec{y}}_i$

$$\begin{aligned} \nabla _{{\varvec{y}}_i}C_2&= 4\sum _j\left( 1-\frac{\delta _{ij}\alpha ^{\prime} + (1-\delta _{ij})\beta ^{\prime}}{O}\right) \\&\quad \cdot q_{ij}\left( 1+d_{ij}^2\right) ^{-1} \cdot ({\varvec{y}}_i-{\varvec{y}}_j). \end{aligned}$$

Finally, we have derivative:

$$\begin{aligned} \nabla _{{\varvec{y}}_i}C&= \nabla _{{\varvec{y}}_i}C_1 + \nabla _{{\varvec{y}}_i}C_2 \\&= 4\sum _j\left( p_{ij} - \frac{\delta _{ij}\alpha ^{\prime} + (1-\delta _{ij})\beta ^{\prime}}{O}\cdot q_{ij}\right) \\ &\quad \cdot (1+\Vert {\varvec{y}}_i-{\varvec{y}}_j\Vert ^2)^{-1}({\varvec{y}}_i - {\varvec{y}}_j). \end{aligned}$$

## Appendix 2: Proof the normalized Laplacian score has range [0, 1]

Given an undirected graph without self-loops ${\mathcal {G}}$ with *n* nodes, and its symmetric adjacency matrix ${\varvec{A}}\in \{0,1\}^{n\times n}$ with zero diagonal, the Laplacian matrix of the graph has form ${\varvec{L}}= {\varvec{D}}- {\varvec{A}}$, where ${\varvec{D}}=diag(sum({\varvec{A}},1))$ is a diagonal matrix with node degrees on the diagonal. We further denote $\tilde{\varvec{A}} = {\varvec{D}}^{-1/2}{\varvec{AD}}^{-1/2}$ and define the normalized Laplacian matrix as $\tilde{\varvec{L}} = {\varvec{D}}^{-1/2}{\varvec{LD}}^{-1/2} = {\varvec{I}}_{n\times n} - \tilde{\varvec{A}}$, where ${\varvec{I}}_{n\times n}$ is a $n\times n$ identity matrix. It is well-known that $\tilde{\varvec{L}}$ is positive semidefinite. Indeed, for any non-zero ${\varvec{x}}\in {\mathbb {R}}^n$, we have

$$\begin{aligned} \frac{{\varvec{x}}^{\prime}\tilde{\varvec{L}}{\varvec{x}}}{{\varvec{x}}^{\prime}{\varvec{x}}}&= \frac{{\varvec{x}}^{\prime}({\varvec{I}}_{n\times n} - \tilde{\varvec{A}}){\varvec{x}}}{\varvec{x}^{\prime}{\varvec{x}}}, \\&= \frac{1}{{\varvec{x}}^{\prime}{\varvec{x}}}\left( \sum _{i=1}^n {\varvec{x}}_i^2 - \sum _{(i,j);{\varvec{A}}_{ij}=1} \frac{2{\varvec{x}}_i{\varvec{x}}_j}{\sqrt{d_id_j}}\right) , \\&= \frac{1}{{\varvec{x}}^{\prime}{\varvec{x}}}\sum _{(i,j);{\varvec{A}}_{ij}=1}\left( \frac{{\varvec{x}}_i}{\sqrt{d_i}} - \frac{{\varvec{x}}_j}{\sqrt{d_j}}\right) ^2\ge 0, \end{aligned}$$

where ${\varvec{x}}_i$ and ${\varvec{x}}_j$ are the *i*th and *j*th element of ${\varvec{x}}$, ${\varvec{A}}_{ij}$ is the element on *i*th row and *j*th column of adjacency matrix ${\varvec{A}}$, and $d_i$ is the degree of node *i*.

Analogously, we can obtain that $\frac{{\varvec{x}}^{\prime}({\varvec{I}}_{n\times n} + \tilde{\varvec{A}}){\varvec{x}}}{{\varvec{x}}^{\prime}{\varvec{x}}} \ge 0$. This allows us to upper bound $\frac{{\varvec{x}}^{\prime}\tilde{{\varvec{L}}}{\varvec{x}}}{{\varvec{x}}^{\prime}{\varvec{x}}}$ for any non-zero ${\varvec{x}}\in {\mathbb {R}}^n$ as follows:

$$\begin{aligned}&\frac{{\varvec{x}}^{\prime}({\varvec{I}}_{n\times n} + \tilde{{\varvec{A}}}){\varvec{x}}}{{\varvec{x}}^{\prime}{\varvec{x}}} \ge 0,\\&\quad \Rightarrow \frac{{\varvec{x}}^{\prime}(-{\varvec{I}}_{n\times n} + \tilde{\varvec{A}}){\varvec{x}}}{{\varvec{x}}^{\prime}{\varvec{x}}} \ge -2\frac{{\varvec{x}}^{\prime}{\varvec{I}}_{n\times n}{\varvec{x}}}{{\varvec{x}}^{\prime}{\varvec{x}}},\\&\quad \Rightarrow \frac{{\varvec{x}}^{\prime}\tilde{{\varvec{L}}}{\varvec{x}}}{{\varvec{x}}^{\prime}{\varvec{x}}} \le 2\frac{{\varvec{x}}^{\prime}I_{n\times n}{\varvec{x}}}{{\varvec{x}}^{\prime}{\varvec{x}}} = 2. \end{aligned}$$

Thus, this establishes the well-known fact that for any undirected graph ${\mathcal {G}}$ and any non-zero real vector ${\varvec{x}}$, it holds that $\frac{{\varvec{x}}^{\prime}\tilde{{\varvec{L}}}{\varvec{x}}}{{\varvec{x}}^{\prime}{\varvec{x}}} \in [0, 2]$.

In the current paper, however, we are only concerned with vectors ${\varvec{x}}$ equal to binary label vectors ${\varvec{f}}_l\in \{0,1\}^n$ with number of ones $n_l={\varvec{f}}_l^{\prime}{\varvec{f}}_l>0$. In this case, the upper bound can be reduced by observing that:

$$\begin{aligned} \frac{{\varvec{f}}_l^{\prime}\tilde{{\varvec{L}}}{\varvec{f}}_l}{{\varvec{f}}_l^{\prime}{\varvec{f}}_l}&= \frac{{\varvec{f}}_l^{\prime}({\varvec{I}}_{n\times n} - \tilde{{\varvec{A}}}){\varvec{f}}_l}{{\varvec{f}}_l^{\prime}{\varvec{f}}_l},\\&= 1 - \frac{{\varvec{f}}_l^{\prime}\tilde{{\varvec{A}}}{\varvec{f}}_l}{{\varvec{f}}_l^{\prime}{\varvec{f}}_l},\\&\le 1, \end{aligned}$$

where we used the fact that $\frac{{\varvec{f}}_l^{\prime}\tilde{\varvec{A}}{\varvec{f}}_l}{{\varvec{f}}_l^{\prime}{\varvec{f}}_l}\ge 0$ since both ${\varvec{f}}_l\ge {\varvec {0}}$ and $\tilde{\varvec{A}}\ge {\varvec {0}}$. Thus, we have established that $\frac{{\varvec{f}}_l^{\prime}\tilde{{\varvec{L}}}{\varvec{f}}_l}{{\varvec{f}}_l^{\prime}{\varvec{f}}_l}\in [0,1]$ for any graph and for non-zero label vector ${\varvec{f}}_l$. Finally, using the observation $\sum _{l \in [0,\ldots ,L]} \frac{n_l}{n} = 1$ it is straightforward to obtain

$$\begin{aligned} \sum _{l \in [0,\ldots ,L]} \frac{n_l}{n} \frac{{\varvec{f}}_l^{\prime}{\varvec{D}}^{-1/2}{\varvec{LD}}^{-1/2}{\varvec{f}}_l}{{\varvec{f}}_l^{\prime}{\varvec{f}}_l} = \sum _{l \in [0,\ldots ,L]} \frac{n_l}{n} \frac{{\varvec{f}}_l^{\prime}\tilde{{\varvec{L}}}{\varvec{f}}_l}{{\varvec{f}}_l^{\prime}{\varvec{f}}_l} \in [0,1]. \end{aligned}$$

## Appendix 3: An example for the normalized Laplacian score

Assume a dataset has six data points. In an embedding ${\varvec{Y}}$, a k-NN graph is constructed with $k=2$. Let us assume the graph has two connected component with each component being a clique. The first component corresponds to data points $\{{\varvec{y}}_1, {\varvec{y}}_2, {\varvec{y}}_3\}$ and the second component corresponds to a data points $\{{\varvec{y}}_4, {\varvec{y}}_5, {\varvec{y}}_6\}$. The adjacency matrix reads:

10 $$\begin{aligned} \begin{bmatrix} 0 &\quad 1 &\quad 1 &\quad 0 &\quad 0 &\quad 0 \\ 1 &\quad 0 &\quad 1 &\quad 0 &\quad 0 &\quad 0 \\ 1 &\quad 1 &\quad 0 &\quad 0 &\quad 0 &\quad 0 \\ 0 &\quad 0 &\quad 0 &\quad 0 &\quad 1 &\quad 1 \\ 0 &\quad 0 &\quad 0 &\quad 1 &\quad 0 &\quad 1 \\ 0 &\quad 0 &\quad 0 &\quad 1 &\quad 1 &\quad 0 \end{bmatrix} \end{aligned}$$

We illustrate the usage of Laplacian score (9) by computing Laplacian score of two label assignments on the data points. Recall the normalized Laplacian score:

$$\begin{aligned} \sum _{l \in \{0,1\}} \frac{n_l}{n} \frac{{\varvec{f}}_l^{\prime}{\varvec{D}}^{-1/2}{\varvec{LD}}^{-1/2}{\varvec{f}}_l}{{\varvec{f}}_l^{\prime}{\varvec{f}}_l}. \end{aligned}$$

First, let us consider data points within each clique have the same label. Namely, ${\varvec{l}}= (0,0,0, 1,1,1)^T$ with label vector ${\varvec{f}}_l$ for specific label *l*: ${\varvec{f}}_0 = (1,1,1,0,0,0)^T$, ${\varvec{f}}_1 = (0,0,0,1,1,1)^T$. The normalized Laplacian score for this label assignment is: 0. This is sensible because there is no label discrepancy in the cliques.

Second, let us consider data points that have with different labels in the cliques. Namely, ${\varvec{l}}= (0,1,0, 1,0,1)^T$ with label vector ${\varvec{f}}_l$ for specific label *l*: ${\varvec{f}}_0 = (1,0,1,0,1,0)^T$, ${\varvec{f}}_1 = (0,1,0,1,0,1)^T$. The normalized Laplacian score for this label assignment is: 0.67. The fact that this Laplacian score is larger than the score obtained in the previous case, indicates that there is a larger label discrepancy in the k-NN graph.

## Appendix 4: Analyzing the clusters in the t-SNE and ct-SNE embeddings

We detail the results obtained by applying the feature ranking procedure on the t-SNE and ct-SNE embeddings as described in Sect. 3.1. The *feature ranking* procedure aims to identify the features that separate the selected clusters in an embedding from the rest. This was done by fitting a linear classifier (logistic regression) on the selected cluster against all other data points. By inspecting the feature importance (classifier weights), we identified the feature with largest contribution. Along with the feature ranking, we further provide the statistics (e.g., mean, standard deviation) of the selected clusters.

### Embeddings of synthetic dataset

In Table 2, feature ranking shows the dimensions with high importance are mainly dimensions 1, 2, 3, and 4. This indicates the embedding in Fig. 1a reveals the clustering structure in dimensions 1–4. The statistics also show all clusters have small variance in dimensions 1–4 while they have larger variance in other dimensions. This further confirms the visualized clusters are from dimensions 1–4.

**Table 2:** Feature importance and statistics of clusters in Fig. 1a

		Dim.1	Dim.2	Dim.3	Dim.4	Dim.5	Dim.6	Dim.7	Dim.8	Dim.9	Dim.10
Purple	Impo.	0.0	0.0	− 7.19	0.22	0.0	0.0	0.0	0.0	0.0	0.0
	Stats.	− 0.50 ± 0.02	− 0.43 ± 0.02	− 1.73 ± 0.03	1.67 ± 0.02	0.01 ± 1.03	0.04 ± 0.99	0.04 ± 0.96	− 0.03 ± 0.96	− 0.05 ± 1.01	− 0.01 ± 0.95
Red	Impo.	0.0	− 3.20	5.73	0.0	0.0	0.0	0.0	0.0	0.0	0.0
	Stats.	− 1.09 ± 0.02	− 1.41 ± 0.02	1.43 ± 0.03	− 0.53 ± 0.02	0.08 ± 1.01	− 0.03 ± 0.99	0.08 ± 1.09	− 0.10 ± 0.97	0.12 ± 0.97	− 0.00 ± 1.07
Orange	Impo.	− 13.62	6.45	− 9.27	− 13.38	− 0.24	− 0.07	− 0.54	0.10	− 0.60	− 0.09
	Stats.	− 0.78 ± 0.03	− 0.33 ± 0.08	− 0.03 ± 0.11	− 0.01 ± 0.05	0.03 ± 0.98	− 0.04 ± 0.99	− 0.04 ± 1.08	0.16 ± 1.02	0.01 ± 1.00	− 0.03 ± 0.97
Green	Impo.	− 0.97	8.06	0.0	1.22	0.0	0.0	0.0	0.0	0.0	0.0
	Stats.	0.57 ± 0.02	1.55 ± 0.02	0.14 ± 0.03	0.24 ± 0.02	− 0.16 ± 0.96	0.03 ± 1.03	− 0.02 ± 0.90	− 0.05 ± 1.04	− 0.12 ± 1.07	− 0.04 ± 1.02
Blue	Impo.	− 5.57	3.12	0.0	0.0	0.0	0.0	0.0	0.0	0.0	0.0
	Stats.	1.63 ± 0.02	0.39 ± 0.02	0.20 ± 0.03	− 1.38 ± 0.02	0.05 ± 1.00	− 0.00 ± 1.00	− 0.05 ± 0.97	0.04 ± 0.98	0.06 ± 0.93	0.09 ± 0.97

In Table 3, feature ranking shows the dimensions with high importance are mainly dimension 5 and 6. This indicates the embedding in Fig. 1b reveals the clustering structure in dimensions 5–6. The statistics also show all clusters have small variance in dimensions 5–6 while they have larger variance in other dimensions. This further confirms the visualized clusters are from dimensions 5–6.

**Table 3:** Feature importance and statistics of clusters in Fig. 1b

		Dim.1	Dim.2	Dim.3	Dim.4	Dim.5	Dim.6	Dim.7	Dim.8	Dim.9	Dim.10
Triangle	Impo.	0.0	− 0.82	0.27	0.08	− 8.35	8.33	− 0.21	− 0.25	− 0.33	0.32
	Stats.	0.03 ± 1.00	0.04 ± 1.01	0.01 ± 0.98	− 0.01 ± 1.0	− 0.91 ± 0.28	0.98 ± 0.30	0.1 ± 1	− 0.06 ± 0.96	− 0.10 ± 0.93	− 0.01 ± 0.89
Circle	Impo.	0.58	− 0.07	0.29	0.21	4.94	5.29	− 0.27	0.54	− 0.36	− 0.49
	Stats.	0 ± 0.99	0 ± 0.99	− 0.04 ± 1.01	0.04 ± 1.02	0.98 ± 0.32	0.99 ± 0.28	− 0.04 ± 1.03	0.15 ± 0.97	− 0.05 ± 1.11	0.06 ± 0.99
Square	Impo.	− 0.48	0.67	0.17	− 0.21	5.20	− 5.55	− 0.47	0.01	0.32	0.02
	Stats.	− 0.03 ± 1.04	− 0.12 ± 0.93	0.04 ± 1.01	− 0.08 ± 1.01	0.97 ± 0.31	− 0.95 ± 0.31	− 0.04 ± 0.98	0.10 ± 1.02	0.08 ± 0.89	− 0.03 ± 1.04
Diamond	Impo.	0.38	0.36	0.16	0.0	− 5.26	− 5.67	0.18	− 0.57	0.0	0.22
	Stats.	0.01 ± 0.97	0.08 ± 1.05	0.00 ± 0.99	0.04 ± 0.97	− 0.90 ± 0.43	− 0.92 ± 0.27	− 0.03 ± 0.95	− 0.15 ± 1.01	0.05 ± 1.04	− 0.02 ± 1.06

Table 4 shows the analysis of a randomly selected local region against equal number of randomly sampled data points in the rest of the population. Feature ranking shows the dimensions with slightly higher importance are mainly dimensions 7–10. This indicates the embedding in Fig. 1c picks up the random noise in dimensions 7–10. The statistics also show relatively small variance in dimension 7–10 while they have larger variance in other dimensions. This further confirms that the visualization is mainly about the random noise in dimensions 7–10.

**Table 4:** Feature importance and statistics of clusters in Fig. 1c

		Dim.1	Dim.2	Dim.3	Dim.4	Dim.5	Dim.6	Dim.7	Dim.8	Dim.9	Dim.10
Random	Impo.	0.004	0.0005	− 0.008	− 0.004	− 0.008	0.002	0.01	− 0.04	− 0.009	− 0.05
	Stats.	− 0.02 ± 1.00	− 0.04 ± 0.98	− 0.03 ± 1.03	0.03 ± 1.02	− 0.00 ± 0.96	− 0.03 ± 1.01	0.11 ± 0.75	− 0.40 ± 0.67	− 0.05 ± 0.64	− 0.45 ± 0.62

### Embeddings of UCI Adult dataset

In Table 5, feature ranking shows the ‘ethnicity’, ‘gender’, and ‘income’ are the features that separate any cluster in Fig. 3a from the rest of the population. The statistics also show all clusters have small variance in the aforementioned three features, while having larger variance in other features. This further confirms the visualized clusters are formed because they have different combinations of features: ‘ethnicity’, ‘gender’, and ‘income’.

**Table 5:** Feature importance and statistics of clusters in Fig. 3a

		Age	Education	Ethnicity	Gender	Hours per week	Income
Hollow circle	Impo.	0.0	0.0	3.84	0.0	0.0	− 5.23
	Stats.	0.44 ± 0.84	0.50 ± 0.87	0.44 ± 0	− 0.42 ± 0.75	0.42 ± 0.85	− 1.73 ± 0
Triangle	Impo.	0.0002	0.0001	− 0.08	0.005	− 0.002	0.004
	Stats.	− 0.05 ± 0.96	− 0.16 ± 0.99	− 2.27 ± 0	0.28 ± 1.05	− 0.15 ± 0.82	0.20 ± 0.85
Full blue circle	Impo.	0.0	0.0	5.14	0.0	0.0	6.28
	Stats.	− 0.16 ± 1.00	− 0.13 ± 0.98	0.44 ± 0	0.08 ± 1.02	− 0.10 ± 1.04	0.58 ± 0
Full orange circle	Impo.	0.0	0.0	3.85	5.68	− 1.76	3.87
	Stats.	− 0.23 ± 1.03	− 0.12 ± 0.83	0.44 ± 0	1.35 ± 0	− 0.46 ± 0.75	0.58 ± 0

In Table 6, feature ranking shows the ‘ethnicity’ and ‘income’ are the features that separate each cluster in Fig. 3b from the rest of the population. The statistics also show all clusters have small variance in the aforementioned two features, while having larger variance in other features. This further confirms the visualized clusters have the ‘gender’ information reduced when comparing to Fig. 3a.

**Table 6:** Feature importance and statistics of clusters in Fig. 3b

		Age	Education	Ethnicity	Gender	Hours per week	Income
Full triangle	Impo.	0.0	− 0.05	− 3.99	0.01	0.0	3.19
	Stats.	− 0.05 ± 1.05	− 0.31 ± 0.97	− 2.27 ± 0	0.37 ± 1.04	− 0.27 ± 0.89	0.58 ± 0
Hollow triangle	Impo.	0.0	0.0	− 5.94	0.0	0.0	0.0
	Stats.	− 0.05 ± 1.05	− 0.31 ± 0.97	− 2.27 ± 0.00	0.37 ± 1.04	− 0.27 ± 0.89	0.58 ± 0.00
Full circle	Impo.	0.0	0.0	5.141	0.0	0.0	6.28
	Stats.	− 0.16 ± 1	− 0.13 ± 0.98	0.44 ± 0	0.08 ± 1.02	− 0.10 ± 1.04	0.58 ± 0
Hollow circle	Impo.	0.0	0.0	3.84	0.0	0.0	− 5.23
	Stats.	0.44 ± 0.84	0.50 ± 0.87	0.44 ± 0	− 0.42 ± 0.75	0.42 ± 0.85	− 1.73 ± 0

In Table 7, feature ranking shows the ‘gender’ and ‘income’ are the features that separate each cluster in Fig. 3c from the rest of the population. The statistics also show all clusters have small variance in the aforementioned two features, while having larger variance in other features. This further confirms the visualized clusters have the ‘ethnicity’ information reduced when comparing to Fig. 3a.

**Table 7:** Feature importance and statistics of clusters in Fig. 3c

		Age	Education	Ethnicity	Gender	Hours per week	Income
Full blue	Impo.	− 3.21	2.17	0.0	− 11.09	3.67	13.53
	Stats.	− 0.15 ± 0.89	− 0.09 ± 0.99	0.04 ± 0.96	− 0.72 ± 0.2	0.15 ± 1.08	0.58 ± 0
Full orange	Impo.	− 0.76	1.54	− 0.38	9.67	− 3.35	8.26
	Stats.	− 0.24 ± 1.00	− 0.16 ± 0.86	− 0.19 ± 1.15	1.35 ± 0	− 0.44 ± 0.72	0.58 ± 0
Full orange	Impo.	0.0	− 0.78	− 1.22	− 3.62	0.0	− 5.96
	Stats.	0.48 ± 0.86	0.45 ± 0.9	0.11 ± 0.88	− 0.68 ± 0.34	0.47 ± 0.86	− 1.73 ± 0
Full orange	Impo.	0.0	0.0	0.0	3.27	0.0	− 2.61
	Stats.	0.04 ± 0.59	0.79 ± 0.75	0.44 ± 0.00	1.35 ± 0.00	− 0.05 ± 0.39	− 1.73 ± 0

In Table 8, feature ranking shows only ‘income’ separates each cluster in Fig. 3d from the rest of the population. The statistics also show all clusters have small variance in the feature ‘income’, while having larger variance in other features. This further confirms the visualized clusters have information other than ‘income’ feature reduced when comparing to other figures.

**Table 8:** Feature importance and statistics of clusters in Fig. 3d

		Age	Education	Ethnicity	Gender	Hours per week	Income
Full orange	Impo.	0.0	0.0	0.0	0.0	0.0	6.50
	Stats.	− 0.14 ± 1.01	− 0.17 ± 0.98	− 0.05 ± 1.05	0.14 ± 1.03	− 0.13 ± 1.02	0.58 ± 0
Full orange	Impo.	0.0	0.0	0.0	0.0	0.0	− 6.01
	Stats.	0.42 ± 0.84	0.50 ± 0.89	0.16 ± 0.83	− 0.41 ± 0.77	0.40 ± 0.83	1.73 ± 0

### Embeddings of DBLP dataset

Table 8 shows the 10 most frequent topics appeared in the clusters in Fig. 5d. The topic words in each line are ordered from the most frequent to the least. The grass green cluster in Fig. 5d consists of papers mainly about ‘clustering’. The purple cluster is mainly about ‘active learning’. The dark green points are papers study ‘data streaming’. The magenta cluster is about ‘privacy’. The orange cluster contains papers that share the topic: ‘computer vision’ (Table 9).

**Table 9:** Feature importance and statistics of clusters in Fig. 5d

	Ten most frequent topics
Grass green	Clustering, algorithm, cure, canopy, cluster, analysis, correlation, k-medians, hierarchical, document
Purple	Learning, active, machine, semi-supervised, algorithm, wake-sleep, multi-task, theory, online, data
Dark green	Data, stream, mining, streams, learning, concept, drift, detection, classification, ensemble
Magenta	Privacy, information, data, software, mining, publishing, social, anonymization, k-anonymity, preserving
Orange	Image, retrieval, segmentation, object, visual, learning, vision, detection, feature, recognition

## Footnotes

1 In t-SNE, perplexity is a user-determined parameter. It is interpreted as a smoothed measure of the number of neighbors around a data point. In order to ensure that the effective neighborhood size of all data points is constant and equal to the perplexity, t-SNE tunes the width $\sigma _i$ of the Gaussian density function for each input data point ${\varvec{x}}_i$, shrinking it when ${\varvec{x}}_i$ is in a dense region, and expanding it when it is in a sparse region.

2 If we set $\alpha <\beta$, ct-SNE will embed the same-labeled data points to be nearby even if they are far apart in the input representation, and differently-labeled data points to be far apart even if they are nearby in the input space. This will result in a low-dimensional representation that confirms the label similarity between the input data points, which differs from the main purpose of the presented work. We would explore the $\alpha <\beta$ setting in our future work.

3 See e.g., https://en.wikipedia.org/wiki/N-body_problem#Other_n-body_problems.

4 These venues are: NIPS, ICLR, ICML, AAAI, IJCAI, KDD, ECML-PKDD, ICDM, SDM, WSDM, PAKDD, VLDB, SIGMOD, ICDT, ICDE, PODS, SIGIR, WWW, CIKM, ECIR.

5 The network consists of 43,346 paper nodes, 63,446 author nodes, 16,150 topic nodes and 20 venue nodes.

6 Node2vec is a graph embedding method that transforms node similarities in a graph into distances in a lower dimensional Euclidean space. To define node similarity, node2vec utilises random walks: nodes that frequently co-appear in a random walk are considered more similar. In Node2vec, parameter *p* controls the probability of revisiting the just-visited nodes and *q* controls whether the random walk is more similar to a breadth-first-exploration or a depth-first-exploration.

7 See “Appendix 2” for a proof sketch.

8 https://mpld3.github.io.

9 In Sect. 2.3 we provide more information on the *n*-body problem.
